# Supplementary material for: Effect of Gelatin Coating and GO Incorporation on the Properties and Degradability of Electrospun PCL Scaffolds for Bone Tissue Regeneration
Source: Polymers (Basel). 2023 Dec 30;16(1):129. doi: 10.3390/polym16010129 (PMC10780398; doi:10.3390/polym16010129)
Supplement: Supplementary file 1 [file polymers-16-00129-s001.zip › polymers-2778375-supplementary.pdf]

polymers

Supplementary Material

# Effect of gelatin coating and GO incorporation on the properties and degradability of electrospun PCL scaffolds for bone tissue regeneration

Carlos Loyo [1,2], Alexander Cordoba [1], Humberto Palza [3], Daniel Canales [4], Francisco Melo [5], Juan F. Vivanco [6], Raúl Vallejos Baier [7], Carola Millán [7], Teresa Corrales [8], Paula A. Zapata [1] *

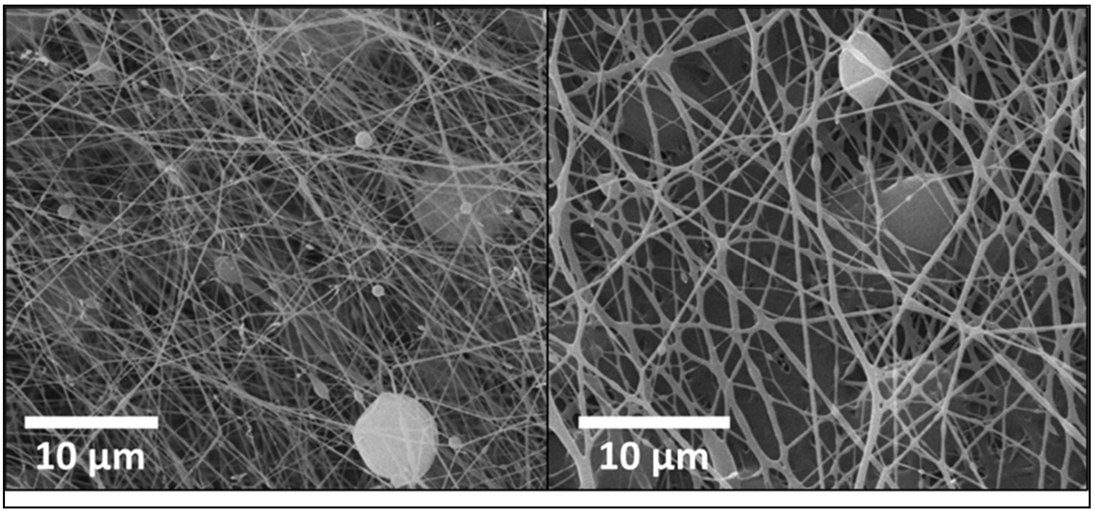


**Figure S1**. Image of SEM of PCL fibers obtained using only chloroform as a solvent.

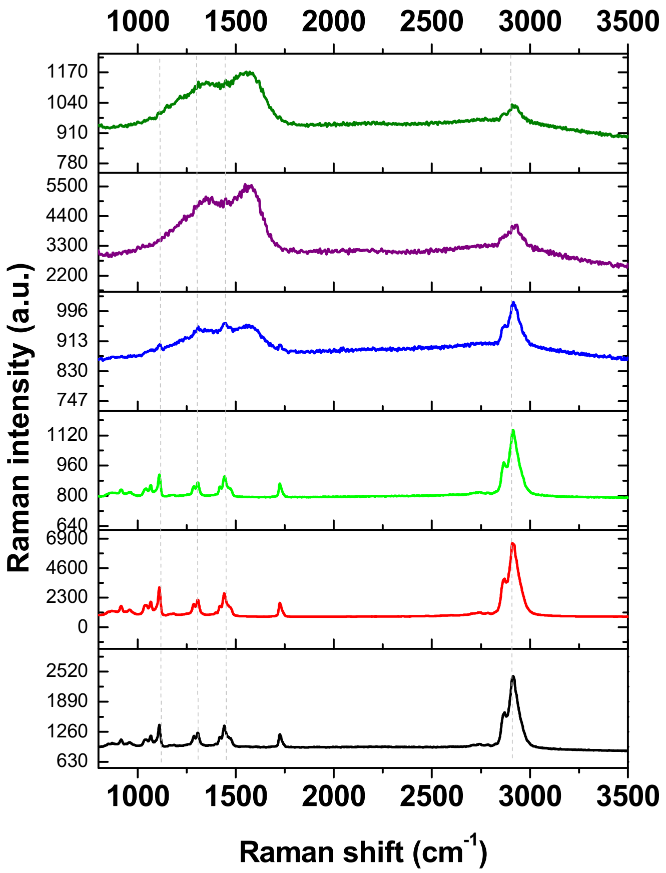


**Figure S2.** Raman intensity vs Raman shift for: PCL, PCL/1 wt%GO, PCL/2 wt%GO, Scaffold with SHINs: PCL/2 wt%GO intermediate spectrum, PCL/1 wt%GO, and PCL/2 wt%GO. (From bottom to top).

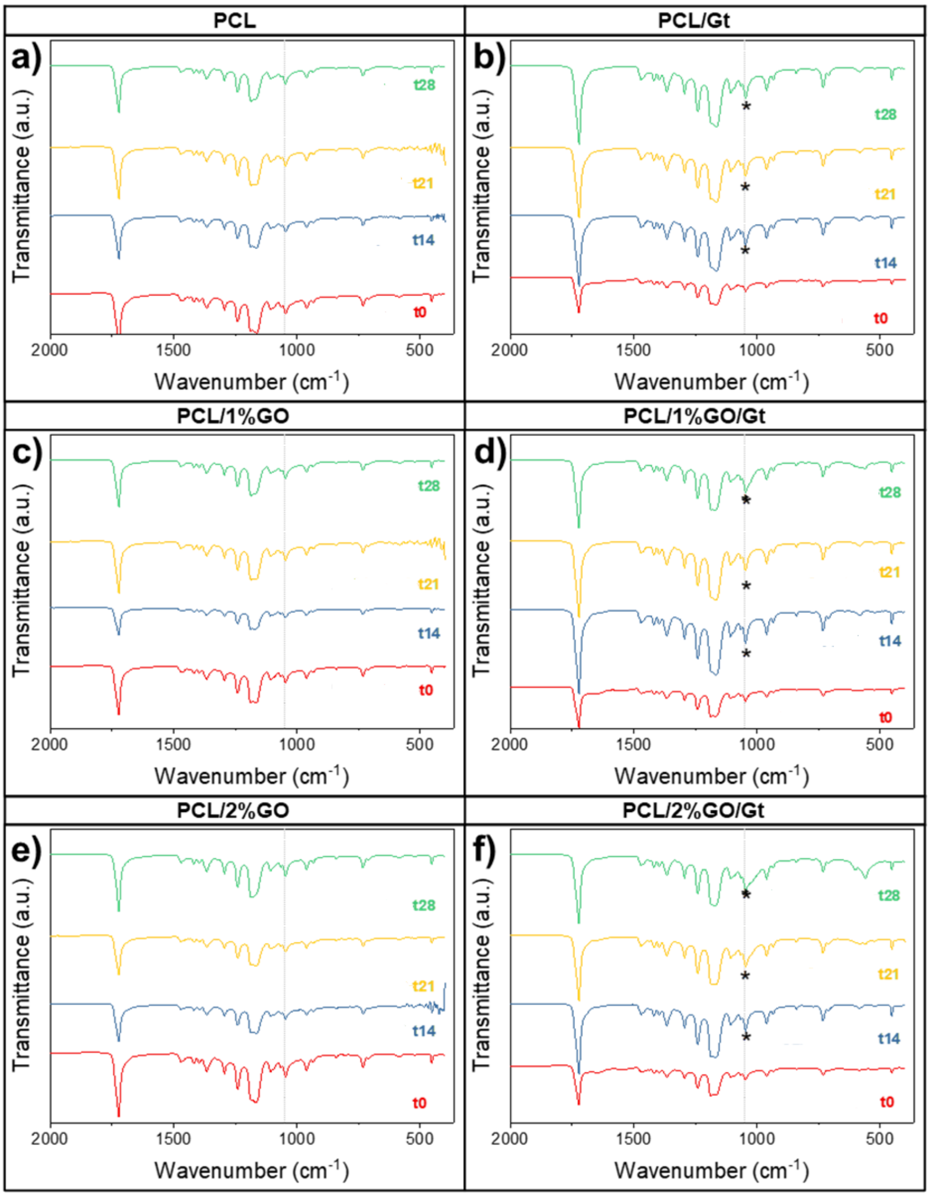


**Figure S3.** ATR-FTIR after 0, 14, 21, and 28 days of bioactivity assay for PCL, PCL/1 wt%GO, and PCL/2 wt %GO scaffolds, and gelatin-coated scaffolds: PCL/Gt; PCL/1 wt %GO/Gt, and PCL/2 wt%GO/Gt.

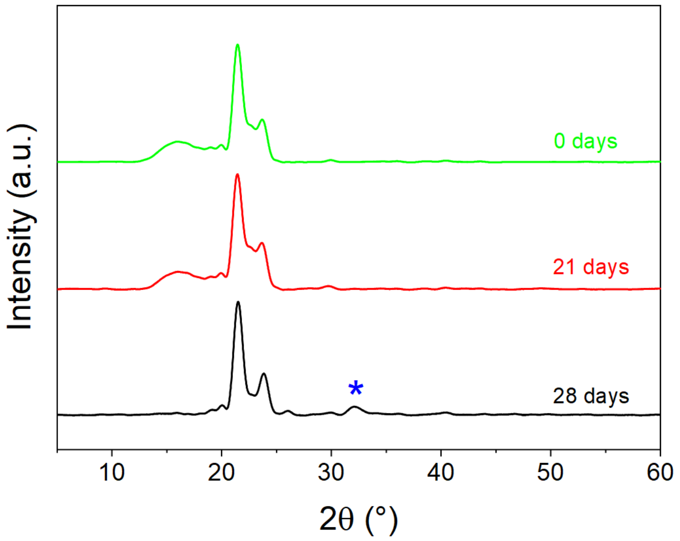


**Figure S4.** XRD after 0, 21, and 28 days of bioactivity assay for PCL/2 wt% GO/Gt.
